# The influence of peer non-suicidal self-harm on young adults’ urges to self-harm: experimental study

**DOI:** 10.1017/neu.2023.51

**Published:** 2023-11-28

**Authors:** Alexandra Pitman, Millie Lowther, Alexandra Pike, Jessica Davies, Angharad de Cates, Joshua E. J. Buckman, Oliver Robinson

**Affiliations:** 1 Division of Psychiatry, UCL, London, UK; 2 Camden and Islington NHS Foundation Trust, St Pancras Hospital, London, UK; 3 UCL Institute of Cognitive Neuroscience, London, UK; 4 Department of Psychology and York Biomedical Research Institute, University of York, York, UK; 5 St Andrew’s Healthcare, Birmingham, UK; 6 Centre for Applied Psychology, University of Birmingham, Edgbaston, Birmingham, UK; 7 Department of Psychiatry, University of Oxford, Warneford Hospital, Oxford, UK; 8 Oxford Health NHS Foundation Trust, Littlemore Mental Health Centre, Oxford, UK; 9 Centre for Outcomes Research and Effectiveness (CORE), Research Department of Clinical, Educational & Health Psychology, University College London, London, UK

**Keywords:** Self-harm, self-injurious behaviour, peer influence, cognition, adolescent

## Abstract

**Objective:**

To test the hypothesis that exposure to peer self-harm induces adolescents’ urges to self-harm and that this is influenced by individual suggestibility.

**Methods:**

We recruited 97 UK-based adults aged 18–25 years with a recent history of self-harm, measuring baseline suggestibility (Resistance to Peer Influence; RPI) and perceived ability to control urges to self-harm (using an adapted item from the Self-Efficacy to Resist Suicidal Action scale; SEASA) before and after two self-harm vignettes featuring named peers from the participant’s social network (to simulate exposure to peer non-suicidal self-harm) and after a wash-out exposure. We used paired *t*-tests to compare mean SEASA scores pre- and post-exposure, and linear regression to test for an association between RPI and change in SEASA scores pre- and post-exposure.

**Results:**

Perceived ability to control urges to self-harm was significantly reduced following exposure to peer self-harm (*t*(96) = 4.02, *p* < 0.001, mean difference = 0.61; 95% CI = 0.31, 0.91), but was not significantly different from baseline after exposure to a wash-out. We found no association between suggestibility and change in urges to self-harm after exposure to peer self-harm.

**Conclusion:**

Our findings support social influences on self-harm in a sample of young adults, regardless of their individual degree of suggestibility.

Significant outcomes

- In a lab setting, risk of self-harm in young people is influenced by peer self-harm, but this is irrespective of individual suggestibility.
- A wash-out exposure neutralised negative effects of exposure to peer self-harm and has potential therapeutic effects in mitigating these risks.
- This is the first experimental study to investigate suggestion effects after self-harm, and findings support those from epidemiological studies.

Limitations

- All those sampled had a history of self-harm, although it is possible that a threshold value of suggestibility is a necessary condition for the onset of self-harm and for susceptibility to peer self-harm influences.
- Findings relate to written vignettes rather than a more naturalistic exposure to peer self-harm.
- Replication of this study is needed in other populations to compare findings.

## Background

Adolescence, spanning the ages 10–24 years (Sawyer *et al*., 2018), is a period during which self-harm is common (Moran *et al*., 2012). Its prevalence is rising among adolescents in high-income countries (Mercado *et al*., 2017; Borschmann & Kinner, 2019; McManus *et al*., 2019; Cybulski *et al*., 2021). Self-harm covers a range of behaviours with varied motivations, reflecting either suicidal intent, non-suicidal distress, or mixed/unclear intentions (Skegg, 2005). People who engage in non-suicidal self-harm (NSSH) describe valuing it emotionally (releasing emotional pressure; expressing self-directed anger) and/or socially (peer conformity; communicating pain) (Klonsky, 2009; Klonsky *et al*., 2015; Edmondson *et al*., 2016). Increasing proportions of adults who self-harm explain it relieves unpleasant feelings of anger, tension, anxiety, or depression (McManus *et al*., 2019). However, self-harm can be stigmatising (Burke *et al*., 2019) and carries a risk of serious tissue damage (Gurung, 2018). Whilst it may have transient ameliorating effects on suicidal ideation (Herzog *et al*., 2022), it reinforces repeat self-harm (Cully *et al*., 2019), and is associated with accidental death (Hawton *et al*., 2006) and suicide (Hawton *et al*., 2015). Where NSSH sets in early as a coping mechanism, it can normalise suicidal behaviour as a response to distress (John *et al*., 2022). The rising incidence of adolescent self-harm could therefore create a cohort effect of young people carrying this theoretical risk of suicide into adulthood (McManus *et al*., 2019).

There is a clear need to address our lack of understanding of the factors driving the rise in adolescent self-harm (Gunnell *et al*., 2018). Theoretical models of self-harm consider the role of exposure to other’s self-harm, social norms and media depictions of self-harm as influencing social cognitions (Hasking *et al*., 2017; O’Connor & Kirtley, 2018). Exposure to peer self-harm is associated with the onset of personal suicidal and non-suicidal self-harm (Jarvi *et al*., 2013; Mueller & Abrutyn, 2015; Quigley *et al*., 2016; Zhu *et al*., 2016), as consistent with qualitative accounts (Hodgson, 2004; Klineberg *et al*., 2013; Hetrick *et al*., 2020; Hall & Melia, 2022). One explanation is that adolescent socialisation processes create pressure to emulate others’ behaviour or conform to social norms, and this may shape susceptibility to peer influences on self-harm and suicidal behaviour (Prinstein *et al*., 2010). Social influences have a greater impact on risk decision-making in adolescence than other age groups, driven by fear of social ostracism (Sebastian *et al*., 2009; Blakemore & Mills, 2014; Knoll *et al*., 2015). Peer conformity rises throughout adolescence (Berndt, 1979; Gardner & Steinberg, 2005; Steinberg & Monahan, 2007; Sumter *et al*., 2009), and the neural correlates of sensitivity to peer influence are apparent in 10-year-olds (Grosbras *et al*., 2007). Adolescence therefore marks a critical developmental period in which there is both greatest susceptibility to peer influence and greatest prevalence of self-harm (Prinstein *et al*., 2010), but also opportunities to intervene.

Social influences on adolescent risk behaviours have been investigated experimentally in relation to driving (Simons-Morton *et al*., 2019) and smoking (Kniskern *et al*., 1983), but to our knowledge, no studies have measured self-harming behaviour before and after manipulating exposure to self-harm (Heilbron & Prinstein, 2008). Understanding how these factors operate in real-time is critical to understanding the temporal sequence of hypothesised suggestion effects; i.e. the effect of a role model’s self-harm or suicidal behaviour on an observer’s self-harming behaviour (Abrutyn & Mueller, 2014). The term suggestion is preferred to contagion or imitation, providing a categorical label for putative mechanisms. Such work is important because identifying individual cognitive markers and peer characteristics influencing susceptibility to self-harm suggestion might inform screening tools in health or educational settings. These could identify individuals who might benefit from targeted interventions after peer self-harm exposure, mitigating suggestion effects. We aimed to test the hypothesis that exposure to peer NSSH induces adolescents’ urges to self-harm, providing evidence to support peer influences on self-harm and that individual suggestibility predicts greater susceptibility to peer influences on self-harm. We also aimed to establish proof-of-concept evidence to support a wash-out exposure achieving neutralising effects.

## Material and methods

### Sample

We recruited adults in late adolescence via social media and research participation databases (see Supplementary Methods). Inclusion criteria were: aged 18–25 years, UK residence, and self-harm within the previous five years. Exclusion criteria were self-reported suicidal ideation or suicide attempt in the past month.

Individuals responding to the advert completed an online screening questionnaire, capturing socio-demographic characteristics and past history of suicidal thoughts, suicide attempts, and self-harm (McManus *et al*., 2019), used for eligibility and risk screening. From June-October 2020 eligible participants (masked to hypotheses) booked an online session lasting 20–30 min, with telephone support from the research assistant on starting and ending.

### Power calculation

We estimated we would need a sample size of approximately 73 participants to detect a difference (between samples paired *t*-test) of two intervals on the 10-point Self-Efficacy to Resist Suicidal Action scale (SEASA) with 80% power and α set at 0.05, calculated using G*Power (Faul *et al*., 2007) version 3.1.9. Anticipating 20% drop-out and data exclusions, we inflated this to 100.

### Ethics

All procedures contributing to this work comply with the ethical standards of the relevant national and institutional committees on human experimentation and with the Helsinki Declaration of 1975, as revised in 2008. All procedures involving human subjects/patients were approved by the UCL Research Ethics Committee (reference 14075/002). Our team’s lived experience researcher advised on project planning and interpretation of results (Lewis & Hasking, 2019). Participants provided online informed consent, including for anonymised data being archived publicly.

### Measures

We used the Gorilla Experiment Builder (www.gorilla.sc) to create and host the experiment (Anwyl-Irvine *et al*., 2019). We collected the following baseline (T0) measures using REDCap electronic data capture tools hosted at UCL (Harris *et al*., 2009, 2019) (validity and scoring details are provided in Supplementary Methods):

- ***Suggestibility:*** (independent variable) Measured using the Resistance to Peer Influence (RPI) Scale (Steinberg & Monahan, 2007), a validated scale that captures suggestibility to peer pressure as distinct from willingness to engage in antisocial activities. Higher scores denote greater resistance to peer influence (low suggestibility).
- ***Perceived ability to control feelings of wanting to self-harm in the next 24 hours:*** (dependent variable) Measured using one item from the original SEASA scale (Czyz *et al*., 2014). Lower scores denote higher risk of imminent self-harm. We adapted the wording to remove references to suicidal intent: ‘*How certain are you that you could control future feelings of wanting to harm yourself*?’.
- ***Socio-demographic characteristics***: Age, gender, occupation, housing status, marital status, and ethnicity.
- ***Personality disorder screen:*** using the Standardised Assessment of Personality – Abbreviated Scale (Fok *et al*., 2015).
- ***Past self-harm or suicide attempt in friends and relatives:*** to derive measures of past exposure to others’ self-harm (none/ lifetime/last year/last week).
- ***Suicide of a friend or relative:*** To derive measures of past exposure to suicide (none/ lifetime/last year/last week).
- ***Catastrophising:*** to capture the contribution of negative cognitive–affective responses to others’ self-harm (Pike *et al*., 2021). Catastrophising is defined as overestimating the probability of a severe negative outcome or perceiving a specific negative event as catastrophic (Pike *et al*., 2021).

### Experimental task

We asked each participant for the first names of three different friends who would feature in a set of three vignettes (two simulating exposure to peer NSSH; one as a wash-out). First, we asked them for the name of someone they admired, then someone they felt ambivalent about, and finally, for our wash-out vignette, we asked them for the name of someone who they enjoyed the company of (see Supplementary Methods for wording). In reporting findings, we term these peers *admired*, *neutral*, and *wash-out,* respectively.

We collected data on nominated peers’ characteristics: age, gender, ethnicity, subjective emotional closeness (using a Likert-style scale from one denoting ‘not close at all’, to five denoting ‘As close as any relationship I’ve had before or since’), perceived likelihood of self-harming in real life (using a Likert-style scale from 0 denoting ‘not likely’ to nine denoting ‘very likely’), and length of the relationship (in years).

**Figure 1.**
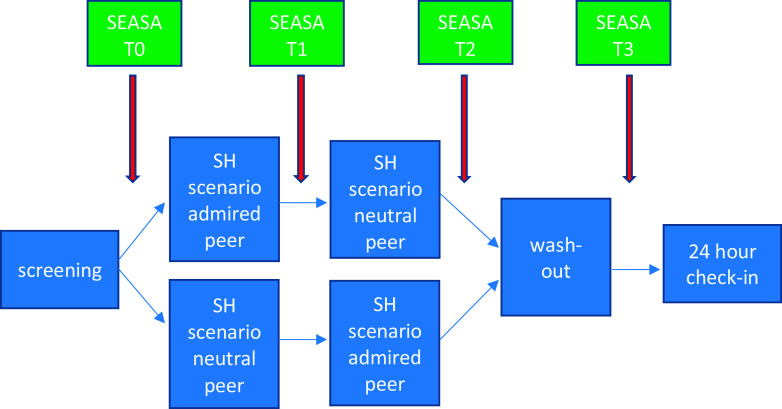
Chronology of tasks and Self-Efficacy to Resist Suicidal Action (SEASA) item measurement.

We then presented each participant with two self-harm vignettes, each describing one of the nominated peers enacting NSSH in response to a life stressor, randomly counterbalanced to present an admired or neutral peer first (Fig. 1).

*“You mentioned earlier that you had a friend called ____________ who you have known for ___years, and who you rated as __ out of 5 on our closeness scale. We would like you to imagine a situation in which you have met up with _______ and at one point in the conversation they mention that things have been difficult recently, and they have been coping with this by self-harming. They don’t describe this to you in any detail but reassure you that they are not suicidal. You get the impression that they have found this helpful in coping with recent problems they have been having.”*

The SEASA item was repeated after each vignette (T1 and T2).

Finally, we presented a wash-out vignette describing a named peer using an app to help them cope with a life stressor. This was intended to neutralise the content presented in the previous self-harm vignettes.

*“You mentioned earlier that you had a friend called ____________ who you have known for ___years, and who you rated as __ out of 5 on our closeness scale. We would like you to imagine a situation in which you have met up with _______ and at one point in the conversation they mention that things have been difficult recently, and they have been coping with this by using an app that helps with anxious thoughts. They tell you about the app and its functions, and explain that it has been really helpful in managing their difficulties. You get the impression that they have found this helpful in coping with recent problems they have been having.”*

Finally, participants repeated the SEASA item (T3).

### Outcomes

Our main outcome was a ***change in perceived ability to control urges to self-harm***, measured as a score difference on the SEASA item (from −9 to + 9) from baseline (T0) to after each self-harm vignette (T1 and T2). We considered score differences to be of more interest than post-exposure score, as the focus of this study was on change from each individual’s baseline.

### Covariates

Multivariate models were adjusted for seven covariates specified *a priori*: age (continuous), gender (seven categories), occupational status (six categories) as a proxy for socio-economic status (Buckman *et al*., 2022), ethnicity (five categories), personality disorder screen (binary), past history of non-fatal self-harm in friends and relatives (categorised on recency), and order of vignette presentation (admired/neutral first). Table 1 specifies categories.

**Table 1.** Demographic and clinical characteristics of study participants

Variable	Low suggestibility^a^ (*n* = 47)		High suggestibility^a^ (*n* = 50)		Total sample (*n* = 97)		*p*-value^b^
	*N*	%	*N*	%	*N*	%	
Age group							
Age 18–21	24	51.0	20	40.0	44	45.4	0.274
Age 22–25	23	48.9	30	60.0	53	54.6	
Missing	0	0	0	0	0	0	
Gender							
Male	8	17.0	4	8.0	12	12.3	0.320
Female	37	78.7	43	86.0	80	82.5	
Transgender female	0	0	0	0	0	0	
Transgender male	1	2.1	1	2.0	2	2.1	
Gender variant/non-binary	0	0	2	4.0	2	2.1	
Other	0	0	0	0	0		
Prefer not to say	1	2.1	0	0	1	1.0	
Missing	0	0	0	0	0	0	
Marital status							
Married	0	0	3	6.0	0	3.1	0.240
Cohabiting	6	12.8	8	16.0	6	14.4	
Single	40	85.1	39	78.0	40	81.4	
Separated	0	0	0	0	0	0	
Divorced	1	2.1	0	0	1	1.0	
Widowed	0	0	0	0	0	0	
Occupational status							
Employed full-time (>20 hours per week)	6	12.8	17	34.0	23	23.7	0.085
Employed part-time (<20 hours per week)	3	6.4	3	6.0	6	6.2	
Full-time home-maker (including caring for young children)	0	0	0	0	0	0	
Full-time carer for parents or other relatives	0	0	0	0	0	0	
Student	29	61.7	19	38.0	48	49.5	
Waiting for a job you have been offered	1	2.1	2	4.0	3	3.1	
Waiting for temporary work	0	0	0	0	0	0	
Temporarily off work (e.g. furloughed; maternity leave; sick leave)	3	6.4	3	6.0	6	6.2	
Unemployed and looking for work	5	10.6	3	6.0	8	8.3	
Unemployed and unable to work due to long-term sickness or Disability	0	0	3	6.0	3	3.1	
Missing	0	0	0	0	0	0	
Housing status							
Home owner	1	2.1	3	6.0	4	4.1	0.051
Tenant	19	40.4	31	62.0	50	51.6	
Living with relative/friend	26	55.3	13	26.0	39	40.2	
Hostel/care home	0	0	1	2.0	1	1.0	
Homeless	0	0	0	0	0	0	
Other	1	2.1	2	4.0	3	3.1	
Missing	0	0	0	0	0	0	
Highest qualifications							
No formal qualifications	1	2.1	0	0	1	1.0	0.495
GCSE(s) or equivalent	4	8.5	7	14.0	11	11.3	
A-Level(s), Scottish Highers, or equivalent	24	51.0	22	44.0	46	47.4	
Vocational qualification, e.g. City and Guilds or HND	1	2.1	3	6.0	4	4.1	
Undergraduate degree, e.g. BA or BSc	12	25.5	9	18.0	21	21.7	
Master’s degree or equivalent higher professional qualification	5	10.6	9	18.0	21	21.7	
Doctorate, e.g. MD or PhD	0	0	0	0	0		
Missing	0	0	0	0	0		
Ethnicity							
Asian	7	14.9	10	20.0	17	17.5	0.656
Black	0	0	1	2.0	1	1.0	
Mixed race	2	4.3	2	4.0	4	4.1	
White	37	78.7	37	74.0	74	76.3	
Other	1	2.1	0	0	1	1.0	
Missing	0	0	0	0	0	0	
Country of residence							
England	44	93.6	44	88.0	88	90.7	0.358
Scotland	3	6.4	4	8.0	7	7.2	
Wales	0	0	2	4.0	2	2.1	
Northern Ireland	0	0	0	0	0	0	
Missing	0	0	0	0	0	0	
Personality disorder screen							
Negative	22	46.8	27	54.0	49	50.5	0.479
Positive	25	53.2	23	46.0	48	49.5	
Missing	0	0	0	0	0	0	
Previous self-harm (suicidal or non-suicidal) of a relative or friend (categorical variable)							
None	2	4.3	4	8.0	6	6.2	0.367
Not sure	3	6.4	1	2.0	4	4.1	
Prior to a year ago	16	34.0	10	20.0	26	26.8	
In the last year	21	44.7	28	56.0	49	50.5	
In the last week	5	5.8	7	14.0	12	12.4	
Missing	0	0	0	0	0	0	
Previous suicide of relative or friend (categorical variable)							
None	34	72.3	29	58.0	63	65.0	0.175
Not sure	0	0	0	0	0	0	
Prior to a year ago	10	21.3	12	24.0	22	22.7	
Within the last year	3	6.4	9	18.0	12	12.4	
Missing	0	0	0	0	0	0	

SEASA item scores	Mean	SD	Mean	SD	Mean	SD	*p*-value^b^
Baseline SEASA item score^c^	7.53	1.52	7.82	1.44	7.68	1.48	0.721
SEASA item score after any self-harm exposure	7.01	2.03	7.13	2.05	7.07	2.15	0.945
SEASA item score after exposure to self-harm of an admired peer	6.75	2.24	7.12	2.33	6.94	2.30	0.425
SEASA item score after exposure to self-harm of a neutral peer	7.28	2.03	7.14	1.95	7.21	2.00	0.782
SEASA item score after wash-out	8.06	1.20	7.94	1.67	8.0	1.47	0.024
Change in SEASA item score between baseline and after exposure to peer self-harm scenarios	0.52	1.40	0.69	1.56	0.61	1.48	0.367

Processing measure	Median	IQR	Median	IQR	Median	IQR	*p*-value^b^
Catatastrophising score	81	67-90	73	65-83	76	65-87	0.865

	***N***	%	***N***	%	***N***	%	*p*-value^b^
Order of exposure							
Admired peer first	26	55.3	28	56.0	54	55.7	0.946
Neutral peer first	21	44.7	22	44	43	44.3	

IQR, interquartile range; SD, standard deviation.

a Median split at a RPI score value of 2.7 (IQR = 2.3–3.1; mean = 2.67; SD = 0.60; range = 1.4–3.9).

b 2-sided significance threshold of *p* = 0.01 for all tests.

c Mean scores on this item in a validation sample of 464 US adults enrolled in a substance use disorder treatment programme were 6.92 (SD = 2.87) for the full sample, and 4.39 (SD = 2.82) for the sub-sample of 103 with current suicidal ideation (Czyz *et al*., 2014).

### Statistical analysis

We pre-registered our analysis plan prior to analysis on the Open Science Framework (OSF), made publicly available on 07/09/21 (https://osf.io/2eq8z/; doi: 10.17605/OSF.IO/2EQ8Z).

SEASA item score differences and RPI were fitted as continuous variables in linear regression models.

We presented descriptive statistics on our main measures as univariate associations, split by high *versus* low suggestibility (RPI dichotomised at the median) for ease of interpretation.

To test **hypothesis 1**, that there was a reduction in the perceived ability to control urges to self-harm after exposure to peer self-harm, we used paired *t*-tests to assess differences in mean SEASA item scores pre- and post-exposure. In a set of hypothesis-generating analyses (see Supplementary Methods), we tested the effect of stratifying *t*-tests by six peer characteristics specified *a priori* (peer’s age, peer’s gender, peer’s ethnicity, a rating of emotional closeness, perceived likelihood of self-harming in real life, and length of the friendship) and by peer status (whether the admired or neutral peer was presented first).

To test **hypothesis 2**, we used multivariate linear regression models to estimate the association between suggestibility scores (RPI, continuous measure; independent variable) and change in urges to self-harm between baseline and after exposure to each peer self-harm vignette (difference in SEASA item scores, continuous measure; dependent variable), adjusted for the seven pre-specified covariates above. Residuals for RPI values were checked to test assumptions for linear modelling. Multilevel models included two measures per individual (one score difference per vignette), clustered on the individual, using robust standard errors. For comparison of findings (to aid interpretability), linear regression models were repeated with a binary exposure (median split).

In our **proof-of-concept analysis** of the effects of a wash-out vignette, we used paired *t*-tests to estimate the mean difference between SEASA item scores: (a) after exposure to the self-harm vignettes (using the mean of scores after exposure to self-harm of the admired peer and of the neutral peer) and after exposure to the wash-out vignette (hypothesising an improvement in perceived ability to control urges to self-harm after wash-out), and (b) after exposure to the wash-out vignette and baseline scores (hypothesising no difference).

To test our **secondary hypotheses** (see Supplementary Methods), we conducted interaction tests by fitting the following terms to our multivariate linear regression models: peer status, participant age, and history of suicide bereavement.

In an additional hypothesis-generating analysis to test the contribution of high catastrophising scores in explaining whether those with a negative cognitive-affective response to others’ self-harm may be more likely to exhibit change in scores post-exposure, we added catastrophising scores to our final linear regression models to assess whether this changed the magnitude or direction of any association.

For all tests, we used a significance threshold of *p* < 0.01 to reflect multiple testing (see Supplementary Methods). All analyses were conducted using Stata 17 software (StataCorp, 2017).

Due to the nature of data collection, there were no missing data for any of the covariates used in models.

#### Sensitivity analyses

In our sensitivity, analyses we explored the effect on our main findings of:

- Excluding participants who nominated friends outside their age group (defined as over 5 years younger or over 10 years older).
- Excluding data on participants with very fast completion times on the task (defined as the bottom quartile for total time taken), assuming that these participants had thought less carefully about the task.
- Conducting analyses using the statistical package R (version 4.0.3) (R Core Team, 2020).

## Results

### Sample characteristics

**Figure 2.**
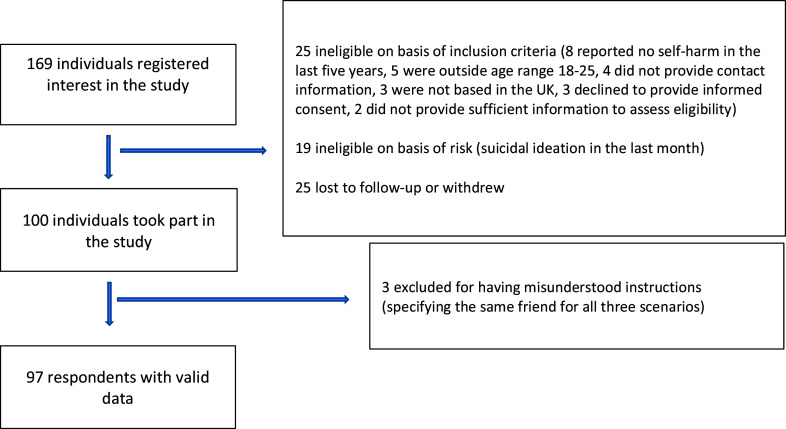
Flow of participants.

Of 169 adults responding to our adverts, 97 met inclusion criteria (Fig. 2). Participants identified predominantly as female (83%), White (74%), and residents in England (91%) (Table 1). Mean age was 21.8 years (SD = 2.29). A high proportion reported having friends or relatives with a history of self-harm (90%), suicide (35%), or both (35%). Baseline suggestibility (RPI) and SEASA item scores were normally distributed. There were no significant group differences on any variable when comparing those with low *versus* high suggestibility scores (based on a median split). The peers nominated for the three vignettes (Supplementary Table 2) ranged in age from 10 to 69 years and were predominantly female (66%).

### Influence of exposure to peer self-harm on urges to self-harm

**Figure 3.**
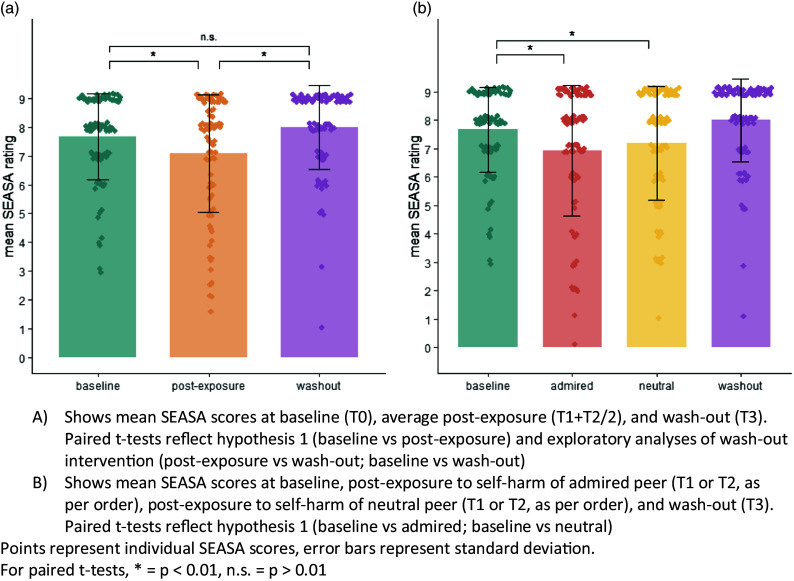
(a) and (b): Mean Self-Efficacy to Resist Suicidal Action (SEASA) item scores at baseline (T0), post-exposure (T1/T2), and wash-out (T3).

We present comparisons of the SEASA item scored at four points: baseline (T0); after first self-harm vignette (T1), after second self-harm vignette (T2), and after wash-out vignette (T3; Table 1; Fig. 3).

In support of **hypothesis 1,** perceived ability to control urges to self-harm decreased significantly between baseline and after: (i) any exposure to a self-harm vignette (mean difference = −0.61; 95% CI = −0.31, −0.91) (Table 2; Fig. 3 left panel); (ii) exposure to an admired peer’s self-harm (mean difference = −0.74; 95% CI = −0.39 to 1.10) (Fig. 3 right panel); and (iii) after exposure to a neutral peer’s self-harm (mean difference = −0.47; 95% CI = −0.17 to 0.78). Supplementary Table 3 presents findings regarding the influence of peer characteristics.

**Table 2.** Results of paired *t*-tests comparing SEASA item scores between baseline, exposure to self-harm vignettes, and wash-out vignette

Comparison†	Baseline (T0) *versus* any self-harm exposure (T1+T2/2)	Baseline (T0) *versus* exposure to self-harm of an admired peer (T1 or T2 as per order)	Baseline (T0) *versus* exposure to self-harm of a neutral peer (T1 or T2 as per order)	Any self-harm exposure (T1+T2/2) *versus* wash-out (T3)	Baseline (T0) *versus* wash-out (T3)
Reference value (mean, SD)	7.68 (1.48)	7.68 (1.48)	7.68 (1.48)	7.07 (2.15)	7.68 (1.48)
Comparison value (mean, SD)	7.07 (2.15)	6.94 (2.30)	7.21 (2.00)	8.0 (1.47)	8.0 (1.47)
Mean difference (95% CI)	−0.61 (−0.31, −0.91)	−0.74 (−0.39, −1.10)	−0.47 (−0.17, −0.78)	0.93 (0.66–1.20)	0.32 (0.05, 0.59)
*t*-statistic	−4.02	−4.16	−3.08	6.86	2.36
*p*-value‡	**0.0001**	**0.0001**	**0.0027**	**0.00001**	0.0204

† Number of observations for all *t*-tests = 97; degrees of freedom for all *t*-tests = 96.

‡ Significance threshold of *p* < 0.01 for all tests.

### Association between suggestibility and changes in urges to self-harm

**Figure 4.**
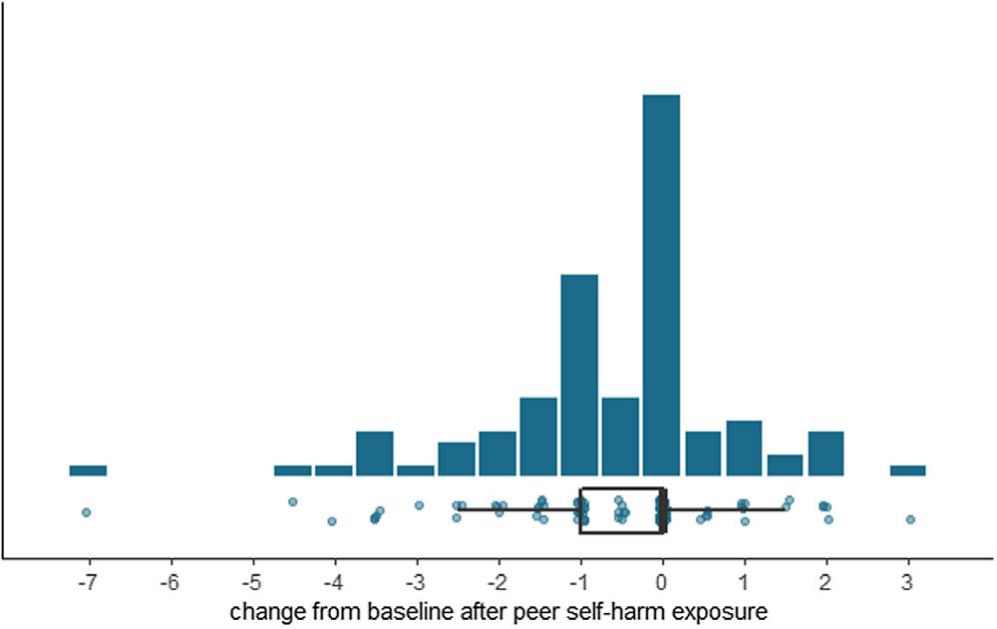
Distribution of change in Self-Efficacy to Resist Suicidal Action (SEASA) item scores between baseline and after exposure to self-harm vignettes.

Change in SEASA item scores between baseline and after exposure to self-harm was normally distributed (Fig. 4). We found no evidence to support **hypothesis 2** regarding an association between suggestibility (RPI, continuous measure) and changes in perceived ability to control urges to self-harm between baseline and after exposure to self-harm (adjusted coefficient = 0.153; 95% CI = −0.556, 0.863; *p* = 0.669), taking into account clustering (Model 4; Table 3). Findings were also non-significant using a binary exposure.

**Table 3.** Association between suggestibility scores and change in perceived ability to control urges to self-harm (n = 97)

Outcome	coefficient	95% CIs	p-value‡
**Change in SEASA item score between baseline and after self-harm exposure (Hypothesis 2)†**			
Model 1: unadjusted	0.047	−0.384, 0.478	0.831
Model 2: adjusted for age, gender, occupation, ethnicity	0.256	−0.309, 0.820	0.371
*Model 3:* as for Model 2, plus possible PD diagnosis, and past exposure to family/friends’ self-harm	0.177	−0.491, 0.845	0.600
*Model 4:* as for Model 3, plus order of presentation of vignettes **(final model)**	0.153	−0.556, 0.863	0.669
*Model 5:* as for Model 4 (final model), plus catastrophising score (hypothesis-generating analysis)	0.091	−0.71, 0.89	0.821

SEASA, Self-Efficacy to Resist Suicidal Action; CI, confidence interval.

† Accounting for clustering based on two measurements per participant (exposed to two self-harm vignettes).

‡ Significance threshold of *p* < 0.01 for all tests.

### Influence of wash-out exposure on urges to self-harm

There was evidence to support our hypothesis of a significant difference between SEASA item scores before and after the wash-out vignette (mean difference = 0.93; 95% CI = 0.66, 1.20), and of no significant difference between SEASA item scores after the wash-out vignette and baseline (mean difference = 0.32, 95% CI = 0.05, 0.59). A high proportion of peers selected for the wash-out vignette were rated as close (93%), similar to ratings for admired peers (95%) but in contrast to those for neutral peers (26%; Supplementary Table 2).

### Secondary hypotheses

There was no evidence to support a modifying effect for any of three variables (peer status, participant age, and history of suicide bereavement) in the association between suggestibility and changes in perceived ability to control urges to self-harm (Supplementary Results), acknowledging under-powered analyses. Adding catastrophising scores to our final model did not influence the magnitude or direction of the non-significant association (Table 3).

### Sensitivity analyses

Our findings were unchanged when repeating our analyses for hypotheses 1 and 2 where: (a) excluding participants completing the task very rapidly, (b) excluding participants who selected peers outside their age group, (c) using R.

## Discussion

### Main findings

We found evidence to support our main hypothesis: exposure to peer NSSH influences adolescents’ urges to self-harm regardless of the peer’s social status. However, findings did not support our hypothesis that more suggestible individuals would demonstrate a more marked inability to control urges to self-harm after self-harm exposure. This suggests that our sample of young adults with a history of self-harm were susceptible to peer self-harm influences regardless of their individual suggestibility, with no grounds for risk stratification based on suggestibility scores. The wash-out exposure we designed for this experiment, modelling alternatives to NSSH, was found to have achieved its aim of restoring self-harm risk to baseline values, thereby neutralising the negative effects of peer NSSH exposure. However, this may have partly relied on peers nominated for this vignette being perceived as close friends.

### Findings in the context of other studies

To our knowledge, this is the first experimental study to investigate suggestion effects after self-harm. Our findings are consistent with longitudinal epidemiological evidence describing an increased risk of self-harm after exposure to peer self-harm, although those analyses captured outcomes at a much longer interval after exposure (Quigley *et al*., 2016). The limited number of studies investigating clustering of self-harm within adolescent communities provide evidence to support clustering effects within psychiatric units (Taiminen *et al*., 1998) but not in schools (Pisinger *et al*., 2019). Our findings from a sample of people who all had a history of self-harm would be consistent with this. There has been little research investigating the influence of perceived social norms of NSSH (including anticipation of social approval/sanctions and misperceptions of others’ NSSH rates) on own NSSH (Dempsey *et al*., 2023), and more work is needed to understand how these modify the effects of direct exposure to peer NSSH. Our findings regarding the wash-out are consistent with evidence that adolescents demonstrate sensitivity to the quality of social information, integrating safe social information into their decision-making (Ciranka & Van Den Bos, 2019). They also accord with findings regarding media influences, finding that exposure to recovery-oriented and hopeful media depictions of self-harm is associated with positive attitudes towards recovery among young people who self-harm (Lewis *et al*., 2018). This highlights the importance of guidelines for the responsible representation of NSSH in the media (Westers *et al*., 2021).

### Strengths and limitations

We conducted a hypothesis-led study, recruiting nationally within the UK with clear inclusion criteria, lived experience involvement, and a robust safeguarding procedure. We recruited sufficient participants to exceed the number needed for adequate power in our main analyses and followed a pre-published analysis plan. Our sampling methods achieved good representation of ethnic minorities; with 76% of our sample identifying as of White ethnicity compared with 82% of the population in England and Wales in 2021 (ONS, 2022). Although our internet-based recruitment approach created potential for broad geographic reach, our strategy failed to attract respondents from Northern Ireland, where there are higher suicide rates than other devolved nations (O’Neill & O’Connor, 2020) but lower rates of adolescent self-harm (O’Connor *et al*., 2014). The age range of our sample was intentionally narrow as there were ethical concerns about recruiting children for a newly designed experiment. However, with appropriate safeguards, studies spanning the full adolescent age range (10–24 years) would be informative in exploring associations with suggestibility, as this decreases steadily in a curvilinear fashion throughout adolescence (Steinberg & Monahan, 2007). The age range of peers nominated by participants was wide, ranging from 10 to 69 years, suggesting that peer influences within young people’s social circles are not restricted to the same age range. However, this might relate to the wording of our prompts to select peers. Eliciting the names of peers for our experimental task was likely to increase its salience to participants. We were unable to account for the contribution of assortative relating; genetic similarities arising from the tendency for young people to select their friends based on similar characteristics (Joiner, 2003; Prinstein *et al*., 2010). Epidemiological studies show that assortative relating only partially explains the association between peer self-harm and own self-harm (Prinstein *et al*., 2010; Randall *et al*., 2015), but further work is needed to investigate the contribution of genetic similarities.

Our definitions of admired and neutral peers were articulated in ways that attempted to avoid revealing our hypotheses and lacked age restrictions. This meant different participants may have interpreted these instructions differently, particularly in identifying a neutral peer. Other markers of peer status would have been helpful in understanding mechanisms. Further research using larger samples is needed to investigate the influence of self-harm suggestion effects of similarities in peers’ socio-demographic and clinical characteristics. It is also possible that participants guessed our hypotheses, creating the potential for response bias. However, minimising interpersonal contact between research assistants and participants was intended to reduce the effect of such demand characteristics. Whilst we used a validated measure of suggestibility, developed to minimise socially desirable responding (Steinberg & Monahan, 2007), we were limited in available measures of propensity to self-harm given the requirement to capture changes over a short timescale. Whilst the SEASA item used was not designed for our specific purpose, it captured change in the expected direction both after exposure to self-harm and exposure to wash-out. As it does not inquire about intent, we cannot infer whether exposure to self-harm increases probability of enacted self-harm. We acknowledge that ceiling and floor effects of the RPI and/or SEASA item may have limited opportunities to detect change.

Our exposure was hypothetical NSSH so our findings may not be generalisable to those exposed to enacted self-harm or suicide. Findings from a sample recruited from the internet and research databases may also not be representative of the wider population of young UK-based adults. Finally, it is possible that our vignettes exposed participants to a generic anxiety-inducing stimulus, such that score changes reflected general arousal rather than specific self-harm exposure. Inclusion of multiple threat scenarios (for example exam failure, relationship breakdown) would have controlled for a threat stimulus, allowing us to test the effects of specific exposure to self-harm, and whether this differs by knowing the person and/or being close to that person. Such a study design could carry the risk of respondents acclimatising to repeated exposure to stressful scenarios (or conversely becoming increasingly distressed), indicating the need for careful use of wash-out exposures between exposures.

### Clinical, policy, and research implications

Our findings support clinical concerns about self-harm suggestion effects within adolescent friendship groups, suggesting that young people may need extra support when exposed to peer NSSH. They may also have implications for unintended effects of group interventions for young people who self-harm (Abou Seif *et al*., 2022). An estimated 39% of British adolescents know a friend who self-harms, rising to 77% for those who self-harm themselves (Mars *et al*., 2014). Identifying who is most at risk within this population is challenging because we did not find suggestibility scores predictive of risk and our sample was restricted to those with prior self-harm. There is clearly a need for primary prevention approaches to mitigate social influences on self-harm in schools, colleges, and other adolescent communities. Our proof-of-concept findings regarding our wash-out exposure suggest that an acceptable co-produced intervention modelling adaptive coping may be therapeutic for adolescents exposed to peer NSSH. This is likely to be more potent in the immediate aftermath, and when featuring relatively admired peers, thereby propagating positive influences on self-harm. One approach is to appeal to an adolescent’s concerns about a friend who self-harms, providing access to psychoeducation regarding alternatives to self-harm when managing urges (Pengelly *et al*., 2008) alongside modelling alternative means of processing distress. This could potentially reduce anxiety about their friend whilst also mitigating self-harm suggestion effects. This direct approach may be preferable to universal screening for risk of self-harm, which is resource demanding, lacks supporting evidence, and has documented adverse effects (Morken *et al*., 2020). More broadly, our findings suggest a need for better awareness of the potential risks of adolescent exposure to peer self-harm, and research identifying appropriate responses and safer ways of managing distress.

Further experimental work is needed to quantify the longevity of the effects of peer influence, identifying the window of opportunity for therapeutic intervention, and the importance of peer status. In our study, the magnitude of the difference in SEASA item scores was greater for an admired than neutral peer, but we did not compare these differences formally, and this needs testing in a larger sample. It is also possible that a threshold value of suggestibility is a necessary condition for the onset of self-harm (and for behavioural responses to peer self-harm). Suggestibility scores in our sample were lower (denoting greater suggestibility) than those for two normative samples of young people not defined by self-harm history (Steinberg & Monahan, 2007; Grosbras *et al*., 2007), and it is possible that our sample recruited were above such a threshold, based on their self-harm history. There is therefore a need to conduct this experiment in a representative sample of adolescents with and without past self-harm. Further work is also needed to understand the cognitive and neural basis for social influences on self-harming behaviour, and how they are modified by perceptions of the index self-harmer (social status, emotional closeness). Such work would explore the relative contribution of psychological processes such as social modelling, emotional contagion, outcome expectancies, cognitive availability of methods, and cultural norms. These advances might also inform our understanding of how peer suicide influences suicidal behaviour, with implications for managing suicide clusters (Hawton *et al*., 2020).

## Conclusions

Our experimental study supported self-harm suggestion effects in older adolescents with a history of self-harm, irrespective of individual suggestibility. Given proof-of-concept evidence that our wash-out exposure neutralises the negative effects of self-harm exposure, further co-produced development is indicated for implementation among young people exposed to peer self-harm. As all participants in our sample had a history of self-harm, our hypotheses require further testing in a larger, representative sample of adolescents.

## Supplementary material

The supplementary material for this article can be found at https://doi.org/10.1017/neu.2023.51
